# 

**Published:** 1891-02-07

**Authors:** 


### NOTICE TO CORRESPONDENTS.

All MS., Letters, Books for Review, and other matters intended for the Editor should be addressed The Editor, The Lodge, Porchester Square, London, W.

The Editor cannot undertake to return rejected M.S., even when accompanied by stamped directed envelope.

### Notice to Advertisers and Subscribers.

All Advertisements, Orders for Copies of The Hospital, and Business Communications should be addressed to The Publisher, *not to the Editor*, The Hospital, 140, Strand, W.C.

The Cost of Subscription, post free, is as follows:—Per week, 1½d.; per quarter, 1s. 8d.; per half-year, 3s. 3d.; per annum, 6s. 6d. Foreign:—Per annum, 8s. 8d.; payable, in all cases, in advance.

### Restrictions on Gifts to Charities.

At a meeting of the Hospitals Association, held in the board-room at Charing Cross Hospital on the 21st ult., Mr. Bristowe, barrister-at-law, read a paper on "The Legal Restrictions on Gifts to Charity." In the subsequent discussion, Mr. Timothy Holmes remarked upon the unequal exemptions from the Statute of Mortmain at present existing, instancing, among hospitals so privileged, Guy's, St. George's, and the Middlesex. The Universities of Oxford and Cambridge also enjoyed complete exemption, and in all the cases where the restrictions of the Act had been suspended, no inconvenience had resulted to the public, and certainly great benefit had resulted to the corporations and institutions themselves. He failed to see the public object in maintaining the principle underlying the Mortmain Act, and he thought that all who had heard Mr. Bristowe's paper could not but agree in that gentleman's view as to the desirability of abolishing the restrictions now obtaining, and he trusted the Association would use its powerful influence in endeavouring to ventilate the matter in Parliament.

Mr. Amherst D. Tyssen, while admitting the unsatisfactory nature of the law as it now stood, thought there was one note of comfort to be heard, in that, notwithstanding the present state of things, they at least knew something of the bearings of the various Acts dealing with the subject, despite their intricate natures. This was evidenced by the fact that a far less number of charity cases were brought before the courts than formerly. Referring to the paper that had been read, he failed to see how Mr. Bristowe's suggestion that property bequeathed to charities should be sold, and the proceeds handed to the particular charity it was to benefit. How could this be done? On whom would the responsibility of doing it fall? In Mr. Bristowe's paper there seemed to be a suspicion that the principle of the Mortmain Act was peculiar to England, but this was not so. Not only Europe, but India, had laws of similar tenour, extracts from which Mr. Tyssen gave to the meeting, and which showed that in some instances—notably that of India—the restrictions as to bequests to charitable bodies were even more stringent than with us. He ventured to maintain that if it was a public benefit to have charitable institutions they should be supported out of the rates.

Mr. Burdett, after complimenting Mr. Bristowe upon his able paper, and acknowledging the great ability that gentleman had brought to bear upon its preparation, briefly traced the progress of charities from the days when religious bodies were looked to by the community for all the benefits it received, and the growth of this system was undoubtedly the origin of the question of the advisability of charities receiving bequests of land. Referring to the present time and speaking on behalf of charities, he believed it was quite an open question whether there were many persons in a position and anxious to bequeath land to a charity at all. To understand the issues involved in the question of the abolition of the Mortmain Act, we ought to be familiar with the state of those countries where they had never prevailed. In Canada, for instance, the municipalities of Roman Catholic cities were the poorest, because the religious bodies held a great amount of the best land, and not only held it, but were entirely free from rates. For his own part, deeply interested as he was in charities and anxious to see the funds of all well managed institutions increased, he believed the law as it stood had had the most wholesome effect. Referring to the view held by the previous speaker on the subject of hospitals being supported out of the rates, he very strongly condemned such a proposal, and speaking from absolute knowledge, ventured to say that anyone warmly supporting the idea could not surely have the least notion of what the consequences of such a step would be. One matter as bearing somewhat directly on the subject of the bequest of land to charities Mr. Burdett urged upon the meeting, and that was the desirability of would-be benefactors giving during their lives. This was a most excellent plan, and was being followed more and more every year by our wealthiest and noblest men.

Mr. Ryan thought that on so important a subject as that before the meeting the Secretary should say a few words, and he could only regret there were not hospital secretaries present. There could be no doubt as to the justice of the view that gifts in lifetime were far better than bequests by will. It was certainly more creditable to the donor to give during life than to leave money to be handed over at death when the bequest could be of no service to the giver. Mr. Ryan claimed that the hospitals had a strong case for exemption. He moved a resolution, which was put to the meeting, inviting the Council of the Association to take whatever steps they might deem fit to secure exemption for hospitals from the action of these Acts.

Dr. Steele, in seconding Mr. Ryan's motion, said they were of course all very deeply interested in hospitals and in hospital finances, and desired to see the restriction of the Mortmain Act removed. It was a most important subject, and one that had been the object of considerable attention by the Parliamentary Committee of the British Medical Association, who a few months ago had sent round to various hospitals asking whether they had lost bequests in consequence of the Act. From the replies it would seem that one or two had. He would be very happy to assist in any movement for the repeal of the Act, and he had much pleasure in seconding Mr. Ryan's motion.

The Chairman entirely supported Mr. Burdett's views on the subject of giving to charities during one's lifetime, and referring to Mr. Bristowe's paper said that for himself, and he ventured to say for all who had listened to it, it had been of very deep interest, an interest arising from the very thorough way in which Mr. Bristowe had thrown himself into the work. He would not, however, advise, as Mr. Bristowe suggested, a movement to obtain a Royal Commission, but nevertheless it was a question of so much moment that it was worth earnest consideration as to what steps should be taken in the matter.

### Scraps and Gleanings.

Dr. Read has been elected physician to Worcester Infirmary in place of the late Dr. Strange.

On January 21st a ball was given in the Leamington Town Hall in aid of the funds of the Warneford Hospital.

Mr. George Palmer, formerly M.P. for Reading, has presented £1,000 to the funds of the Royal Berks Hospital.

Small-pox is still spreading in Copenhagen, having broken out in another asylum. No fatal cases have yet occurred.

The chapel at the Royal Infirmary, Newcastle-upon-Tyne, which has been redecorated, was reopened on Sunday, January 18th.

We regret to record the death of Major Terry, who for the past few years has filled the post of secretary to the Royal Hants County Hospital.

The retirement of Mr. Sydney Jones, senior surgeon, was announced at the special Quarterly Court of Governors of St. Thomas's Hospital, held on February 4th.

Sir Algernon Borthwick will preside at the festival dinner on May 11th, to commemorate the 20th anniversary of the foundation of the Chelsea Hospital for Women.

The Earl of Derby will preside at a festival dinner in aid of the National Hospital for the Paralysed and Epileptic, Bloomsbury, at the Hotel Métropole, on April 23rd.

The Duke of Clarence and Avondale has consented to preside at a festival dinner in aid of the funds of the Royal Albert Orphan Asylum, Bagshot, to be held at an early date.

A very successful concert and entertainment was given in Bradford, with the object of raising funds for purchasing a piano for use in the wards of the Bradford Children's Hospital.

A concert was given at the Highbury Athenæum on January 30th, under the direction of Mr. W. H. Thomas, in aid of the Ladies' Building Fund of the Great Northern and Central Hospital.

The balance sheet for 1890 of the Yeovil Hospital shows that the expenditure for the year amounted to £446. After paying this and investing £100 in the purchase of Consols the treasurer has a balance in hand of £127 0s. 11d.

The concert at the Brompton Hospital on January 27th was given under the direction of Mrs. Edmeston. The programme included vocal and instrumental music by Mrs. Edmeston, Miss Elliot, M. Tivadar Nachèz, and several others.

The inmates of Croydon Infirmary had their annual treat on Wednesday, January 14th. The programme was long and varied, and included some conjuring tricks performed by Signor Federico, and a ventriloquial sketch by M. de Layen, which were highly appreciated by the patients.

Mr. William Agnew sent a letter, to be read at the meeting of the subscribers and friends of the Pendlebury Children's Hospital, stating that he was willing to forward a cheque for £1,000 to the treasurer as soon as he had been authorised to receive it for the endowment of a cot.

The annual meeting of the City Dispensary was held in the Dispensary Watling Street, Mr. F. P. Alliston presiding. The report, which was adopted, stated during 1890 11,865 patients had received the benefits of the institution. Of these as many as 10,848 had been discharged, cured or relieved.

The annual report of the Bath Eye Infirmary shows a striking increase in number of applications for treatment as compared with those in 1889. The internal management of the infirmary has been conducted with strict economy, the total expenditure for the year being £470 12s. 1d.

The Queen has sent 95 lbs. of cast linen to the Cancer Hospital, Brompton; ten brace of pheasants to the Royal London Ophthalmic Hospital; ten brace of pheasants to the City Hospital for Diseases of the Chest; 240 lbs. of cast linen and ten brace of pheasants to the Great Northern Central Hospital.

The speakers at the meeting of the supporters of the Manchester Royal Eye Hospital were able to show that the institution not only receives encouraging support, but that great care and economy are displayed in its management, while the efficiency of the work is maintained.

The annual meeting of the Governors of the Royal Maternity Society was held on January 27th, Mr. C. Barham in the chair. Mr. J. Whittington moved the adoption of the report, and said there was much cause for congratulation in the report of a society which was now 134 years of age. The work of the charity ought to be more widely known. The report was adopted.

During 1890 there were some unusually severe cases amongst the in-patients of the Tiverton Infirmary and Dispensary. Several operations were performed, the results in all cases being satisfactory. The financial [illegible]port for the year shows that the receipts amounted to £663 9s. 9d., and the expenditure to £637 6s. Great credit is due to Miss Reilly, the matron, for her rigid economy.

The most remarkable feature in the financial report of the Brighton, Hove, and Preston Dispensary is the generosity displayed with regard to the Western Branch. At the beginning of the year 1890 there remained a debt on the building of £709 5s. 11d. To meet this sum £466 6s. 4d. was raised by a bazaar, and £100 and £15 15s. were sent respectively by two unknown donors.

The annual meeting of the Liverpool Infirmary for Children was held under the presidency of the Mayor. The thirty-ninth annual report stated that there had been 1,216 in-patients and 38,776 out-patients during the year. Mr. F. W. Agnew, in proposing a vote of thanks to the committee, made reference to the sad fact that the death-rate statistics in Liverpool showed that one-fourth of all the deaths were of children under one year of age.

On January 15th a cottage hospital was opened at Goole, where such an institution has long been desired. The building, which was formerly the Court House, was presented by Mr. W. H. Bartholomew, and the cost of altering and furnishing it to suit the requirements of a hospital was borne by the Aire and Calder Navigation directors. Towards the endowment and maintenance funds £2,311 2s. 1d. has been raised, and the committee have promises of annual subscriptions amounting to £200 or more.

### The Student of Medicine.

[All communications for this Supplement should be addressed to The Editor of The Hospital, at 140, Strand, with the words, "Students' Column," written in the left hand top corner of the envelope.]

### APPOINTMENTS.

At the Middlesex Hospital, C. Ernest Soulby, L.R.C.P.Lond., M.R.C.S., L.S.A., and Robert Stanley Thomas, M.A., M.B., B.C.Cantab., have been appointed house surgeons. At the Royal Free Hospital, Ernest Solly, M.B.Lond., F.R.C.S., has been appointed senior resident medical officer. At St. Luke's Hospital for Lunatics, E.C., W. J. Haslett, M.R.C.S., L.R.C.P.Lond., has been appointed resident clinical assistant. At the Edinburgh Royal Maternity and Simpson Memorial Hospital, D. B. Hart, M.D.Edin., F.R.C.P., has been appointed medical officer. G. B. Marshall, M.B., C.M.Edin., has been appointed house surgeon. At the Seamen's Hospital, Greenwich, Matthew Henry Spencer, M.A., M.B., C.M.Camb., has been appointed resident house physician. At the City Asylum, Birmingham, Philip E. W. MacAdam, L.R.C.P., and S.Irel., has been appointed clinical assistant. At the Manchester Royal Infirmary, T. N. Kelynack, M.B., Ch.B., has been appointed pathological registrar. At the County Asylum, Rainhill, Edinburgh, W. G. W. Sanders, M.B., C.M.Edin., has been appointed pathological assistant medical officer.

### VACANCIES.

The following vacancies are announced this week, the amount of salary in each case being given after the name of the institution:

### Resident Medical Officers.

Addenbrooke's Hospital, Cambridge, House Physician—Salary £65 per annum, with Board, &c.
Chichester Infirmary, Chichester, House Surgeon—Salary £80, with Board, &c.
City Hospital for Infectious Diseases, Newcastle-upon-Tyne. Medical Assistant—Salary for first year £50, and if re-appointed £70 for second year, with Board, &c.
City of London Hospital for Diseases of the Chest, Victoria Park, E., House Physician.
City of London Lunatic Asylum, Dartford, Kent, Assistant Medical Officer—Salary £150, with Board, lodging, &c.
Dorset County Hospital, Dorchester, House Surgeon—Salary £70.
East Suffolk and Ipswich Hospital, Assistant House Surgeon—Salary £20, with Board, &c.
Hospital for Sick Children, Great Ormond Street, Bloomsbury. W.C., Resident Medical Officer as House Physician for one year—Salary £60, with Board, &c.
Huddersfield Infirmary, Junior House Surgeon—Salary £40, &c.
National Sanatorium for Consumption and Diseases of the Chest, Bournemouth, Clinical Clerk for four months—Board, &c.
Nottingham General Hospital, Resident Medical Assistant for six months—Board, &c. Also Resident Surgical Assistant—Board, &c.
Southern Hospital, Clifford Street, Manchester, House Surgeon.
General Hospital, Birmingham, Assistant House Surgeon.
Central London Opthalmic Hospital, Gray's Inn Road, House Surgeon.
Central London Sick Asylum District, Assistant Medical Officer and Dispenser for the Asylum in Cleveland Street—Salary £100, with Board, &c.

### Non-Resident Medical Officers.

Chelsea Hospital for Women, Fulham Road, S.W., Physician to the Out-patients.
Farringdon General Dispensary and Lying-in Charity, 19, Bartlett's Buildings, Holborn, E.C., Honorary Physician.
London Fever Hospital, Liverpool Road, N., Physician and Assistant Physician.
National Hospital for the Paralysed and Epileptic (Albany Memorial), Queen Square, Bloomsbury, Surgeon.
St. Mark's Hospital for Fistula and other Diseases of the Rectum, City Road, London, E.C., Anæsthetist—Honorarium, £50 per annum.
Royal Academy of Arts, Professorship of Anatomy.

### PRESENTATION.

Dr. T. N. Kelynack, on leaving Crumpsall Hospital to take up his new duties as Pathologist to the Manchester Royal Infirmary and Assistant Lecturer and Demonstrator in Pathology at the Owens College, was presented by his nurses with an elegant little timepiece and tastefully illuminated address.

### PASS LISTS.

### University of Glasgow.

The following is a list of gentlemen who have received the degrees of M.B. and C.M. since the general graduation in July, 1890: Thomas Primrose Anderson, Partick, Glasgow; Alexander Andrew, Tarbolton, Ayrshire; Alexander Percy Agnew, Burnley, Lancashire; Thomas Berry, Langside, Glasgow; James Cordiner, M.A., Lesmahagow,

### The Hospital Advertiser.

THE HOSPITAL FOR SICK CHILDREN,

GREAT ORMOND STREET, BLOOMSBURY, W.C.

**Pass Lists—(*continued*).**

Lanarkshire; George Henry Edington, Hillhead, Glasgow; Malcolm Gillies, Easdale, by Oban; James Kirkwood, Greenock; Hugh Lang, Woodside, Cardross, Dumbartonshire; George Lowson, Dundee; Arthur Henry Lucas, Carmyle, near Glasgow; Thomas Dryden Moffat, Glasgow; Robert James Nevin, West Auckland, county Durham; Douglas Wills Russell, Paisley: William John More Slowan, Hilton, Kilmalcolm, Renfrewshire; James Sim Wallace, B.Sc., Shawlands, Glasgow; James Alexander Wilson, Langley Moor, Durham; John Zuill, M.A., Buchlyvie, Stirling.

### EVENTS FOR THE MONTH OF FEBRUARY.

### Students' Medical Societies.

St. Bartholomew's Hospital Abernethian Society.—February 12th, Mr. H. J. Waring, B.Sc., on "Moveable Kidney"; February 19th, Mr. H. M. Bowman, M.B., and Mr. H. G. Cook, on "Rheumatic Hyperpyrexia; February 26th, Dr. Cautley, M.R.C.P., on "Headache."

St. George's Hospital Hunterian Society.—February 13th, Dr. Champneys (subject not announced); February 27th, Dr. Slater, on "Antisepsis."

Guy's Hospital Pupils' Physical Society.—February 7th, Debate, "That Inguinal Colotomy is preferable to Lumbar Colotomy in Obstruction of the Rectum and Large Intestine," proposed by F. W. Hall; February 21st, T. R. Taylor, B.Sc., on "Protoplasm."

St. Mary's Hospital Medical Society.—February 18th, Zittman's Treatment of Syphilis, Mr. P. J. Kingston. Clinical cases.

Middlesex Hospital Medical Society:—February 12th, Histological Subjects, Genito-Urinary System, Urine. February 26th. Eye.

St. Thomas' Hospital Medical and Physical Society.—February 12th, Mr. Balmance and Dr. Sherrington on "Inflammation" with Lime-light demonstrations. February 19th, Clinical and Pathological evening.

University College Medical Society.—February 11th. Paper by T. L. Pennell, B.Sc., L.S.A. Vis Medicatrix Naturæ. Pathological Diseases of the Skin: Histological, The Connective Tissues. Chairman J. R. Bradford, M.D., D.Sc. February 25th, Section Mounting under superintendence of Pathology and Physiology Sub-Committees. The Vice-President.

Westminster Hospital Guthrie Society.—February 12th.

### Athletics.

Football Matches.

St. Bartholomew's Hospital.—February 7th, v. London Welsh, at Balham; 14th, v. Croydon, at Croydon; 21st, v. Marlborough Nomads, at Surbiton; 28th, v. Old Paulines, at Chiswick. Second team: February 7th, v. Guy's Hospital, at Raynes Park; 14th, v. Croydon, at Balham; 28th, v. Middlesex Wanderers, at Balham.

Guy's Hospital.—Rugby: first fifteen, February 7th, England v. Ireland, in Ireland; 14th, v. Clapham Rovers, at Wandsworth; 21st, North v. South, at Newcastle, also on 21st v. R.M.C., Sandhurst, at Yorktown; 28th, v. London Scottish, at Richmond. Second fifteen, February 7th, v. St. Bartholomew's second, at Raynes Park; 14th, v. Middlesex Wanderers; 21st, v. St. Thomas's second, at Raynes Park; 28th, v. Clapham Rovers, at Wandsworth. Association: First eleven, February 4th, v. Royal School of Mines, at Raynes Park; 7th, v. Old Wilsonians, at Denmark Hill; 14th. v. Beckenham, at Beckenham; 21st, v. Watford Rovers, at Watford; 28th, v. Walton-on-Thames, at Walton.

King's College.—Association: February 7th, v. Hoddesdon, at Broxbourne; 11th, v. Clapham Rovers, at Wandsworth Common; 28th, v. R.I.E.C., at Cooper's Hill. Rugby: February 7th, v. Kingston Rangers, at Bushey Park; 21st, v. Hampstead, at Neasden; 28th, v. St. Mary's Hospital, at Balham. Second fifteen, February 11th, v. Thomas's third fifteen, at Wormwood Scrubbs.

London Hospital.—Rugby: February 7th, v. Wasps, at home; 21st, v. Sidcup, at Sidcup; 28th, v. Moor Park Wanderers, at Honor Oak. Second fifteen, February 14th, v. Lewisham Park, at home. Association: February 7th, v. Upton Ivanhoe, at Upton Park; 14th, v. Bowes Park, at Bowes Park; 21st, v. Reigate Priory, at Reigate; 28th, v. Foxes, at Edmonton.

St. Mary's Hospital.—Rugby: first fifteen, February 7th, v. Bedford, at home; 11th, v. Royal School of Mines, at home; 21st, v. Dental Hospital, at home. Second fifteen, February 7th, v. University College School, at Willesden Green. Association: February 4th, v Watford Rovers, at Wimbledon; 11th, v. Casuals, at Tooting; 21st, v. Barnes, at Barnes.

Middlesex Hospital.—First fifteen, February 7th, v. Brighton, at Brighton; 14th, v Saracens, at Walthamstow; 11th, v. Bedford, at Bedford; 28th, v. Swindon Rangers, at Swindon. Second fifteen, February 21st, v. Bartholomew's Hospital, at Balham; 28th, v. Lewisham, at Lewisham.

St. Thomas's Hospital.—Association: February 12th, v. Cooper's Hill, at Egham; 14th, v. Newbury, at Newbury; 21st, v. St. George's School, at Harpenden; 28th, v. Lancing Old Boys, at Surbiton; 24th, v. Forest School, away.

University College and Hospital.—First team. February 7th, v. Clapham Rovers, at Neasden; 14th, v. Plaistow, at Plaistow; 21st, v. Old Salways, at Neasden; 28th, v. Crystal Palace Engineers, at Balham. Second team, February 7th, v. St. Margaret's Athletic Club, at St. Margaret's Road.

Westminster Hospital.—February 7th, v. Old Paulines, at Chiswick Park; 14th, v. Central Institute, at Raynes Park; 21st, v. Eltham House, at Raynes Park; 28th, v. 1st Surrey Rifles, at Camberwell.

Owen's College.—February 12th, v. Yorkshire College, away; 18th, v. Salford, at home; A Team, February 7th, v. Broughton Park, at home; 14th, v. Horwich, at home; 21st, v. Mayfield, at home; 28th, v. Free Wanderers (first), at home. B Team, February 7th, v. Broughton Park (A), away; 21st, v. Mayfield (A), away; 28th, v. Free Wanderers, second, away.

